# An Undifferentiated Primary Mediastinal Carcinoma Compressing the Main Pulmonary Artery: A Rare Cause of Right Ventricular Strain

**DOI:** 10.7759/cureus.52789

**Published:** 2024-01-23

**Authors:** Ali Khreisat, Tanya Amal, David M Howell, Steven Timmis

**Affiliations:** 1 Internal Medicine, Corewell Health William Beaumont University Hospital, Royal Oak, USA; 2 Internal Medicine, Beaumont Health, Royal Oak, USA; 3 Internal Medicine, Oakland University William Beaumont School of Medicine, Royal Oak, USA; 4 Cardiovascular Medicine, Corewell Health William Beaumont University Hospital, Royal Oak, USA; 5 Cardiology, Beaumont Health, Royal Oak, USA

**Keywords:** primary mediastinal tumor, right ventricular strain, pulmonary artery compression, anterior mediastinal mass, right ventricular failure, undifferentiated carcinoma

## Abstract

Undifferentiated carcinoma (or poorly differentiated carcinoma) of the mediastinum is a relatively rare pathological variant of anterior mediastinal tumors. Pathologists usually use the term to describe an epithelial tumor with no histological features that enable the identification of its site of origin. Invasion of adjacent vital cardiopulmonary structures is among the most problematic complications of anterior mediastinal masses. We report a case of a 60-year-old male presenting with easy fatiguability, significant weight loss, and chest pain. A CT scan of the chest revealed a large anterior mediastinal mass, compression of the main pulmonary artery, and a large pericardial effusion. The patient underwent pericardiocentesis, emergent radiotherapy, and platinum-based chemotherapy. His condition dramatically improved, and he was subsequently discharged home for further follow-up.

## Introduction

The most common types of anterior mediastinal masses are thymomas, lymphomas, germ cell tumors, congenital cysts, and retrosternal goiter [1]. Approximately 10-15% of anterior mediastinal tumors are characterized as undifferentiated carcinoma based on tissue biopsy [2,3]. Thymic malignancies and retrosternal goiter account for 66% of anterior mediastinal masses in both men and women aged over 40 years. Various rare tumors, including thymolipoma, malignant pleural mesothelioma, and angiosarcoma, comprise only 5-20% of anterior mediastinal masses [4]. 

Anterior mediastinal masses with mass effect upon the pulmonary artery are even more scarce, with only a few cases documented so far, including non-small cell squamous cell carcinoma and pericardial mesothelioma [5,6]. In young adults, acquired pulmonary artery compression is mainly reported in tumors such as teratomas and lymphomas [7]. We present an intriguing, complex case of a 60-year-old male with anterior mediastinal undifferentiated carcinoma, initially complicated by pericardial invasion, massive pericardial effusion, and tamponade. After undergoing pericardiocentesis, right ventricular (RV) strain related to malignancy-acquired pulmonary artery compression was diagnosed.

## Case presentation

The patient was a 60-year-old Caucasian male with a history of HIV infection on antiretroviral therapy and chronic inactive hepatitis B. He presented to the hospital with a week-long history of central, non-radiating chest pain, preceded by unintentional weight loss, night sweats, and general weakness for a month. His vital signs on presentation were within normal limits. Physical examination revealed jugular venous distention and distant heart sounds. Chest X-ray showed a widened mediastinum and enlarged cardiac silhouette. Basic laboratory workup including complete blood count (CBC), renal function panel (RFP), and liver function tests (LFTs) were within normal limits. The initial echocardiogram (Video 1) showed a large, predominately posterior pericardial effusion and normal RV function.

**Video 1:** Short-axis transthoracic echocardiogram The video shows a large pericardial effusion present anteriorly and posteriorly

**Figure 1:**
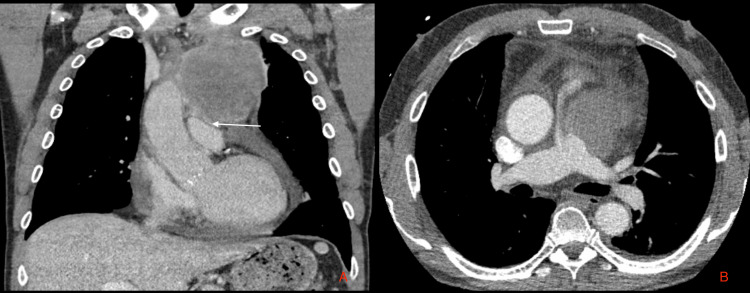
Chest CT of the patient A. Intravenous contrast coronal view showing a 10-cm anterior mediastinal mass with pericardial invasion (white arrow). B. Transverse view showing partially compressed main pulmonary artery by irregular mediastinal mass CT: computed tomography

**Figure 2:**
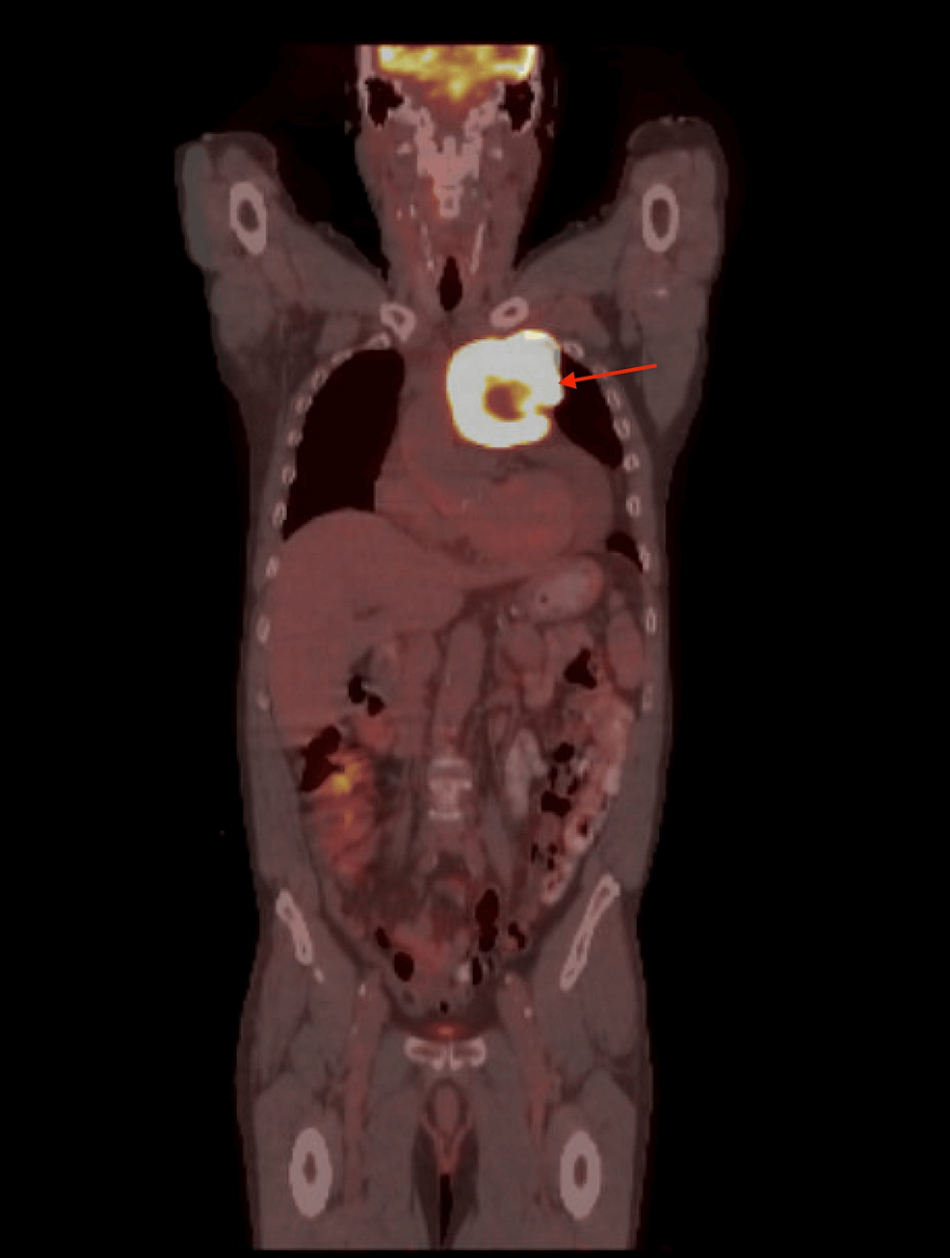
PET scan of the patient The image shows a large FDG-avid left anterior mediastinal mass with increased FDG activity peripherally with max SUV of 24.0 (red arrow) and decreased activity centrally suggestive of malignancy with central necrosis FDG: fluorodeoxyglucose; PET: positron emission tomography; SUV: standardized uptake values

The patient was admitted to the cardiac ICU for hemodynamic monitoring. Because of the posterior-predominant location of his pericardial effusion, pericardiocentesis was challenging, and hence our team considered proceeding with a pericardial window instead. A preoperative CT scan of the chest was performed to delineate the pericardium further. It showed a 10-cm anterior mediastinal mass with pericardial invasion (Figure 1A). PET scan showed a large left anterior mediastinal mass with high fludeoxyglucose (FGD) uptake peripherally and decreased FGD activity centrally, suggestive of central necrosis (Figure 2). Laboratory workup showed no signs of tumor lysis syndrome, mildly elevated lactate dehydrogenase (LDH) at 241 U/L (reference range: 100-240 U/L), carbohydrate antigen 19-9 at 145 u/ml (reference range: <36 u/ml), and normal alpha-fetoprotein and human chorionic gonadotropin levels. He underwent a CT-guided biopsy of the mediastinal mass. Pathology revealed poorly differentiated carcinoma that was p40-negative and claudin-4- and AE13-positive, suggesting a lung-related etiology.

**Figure 3:**
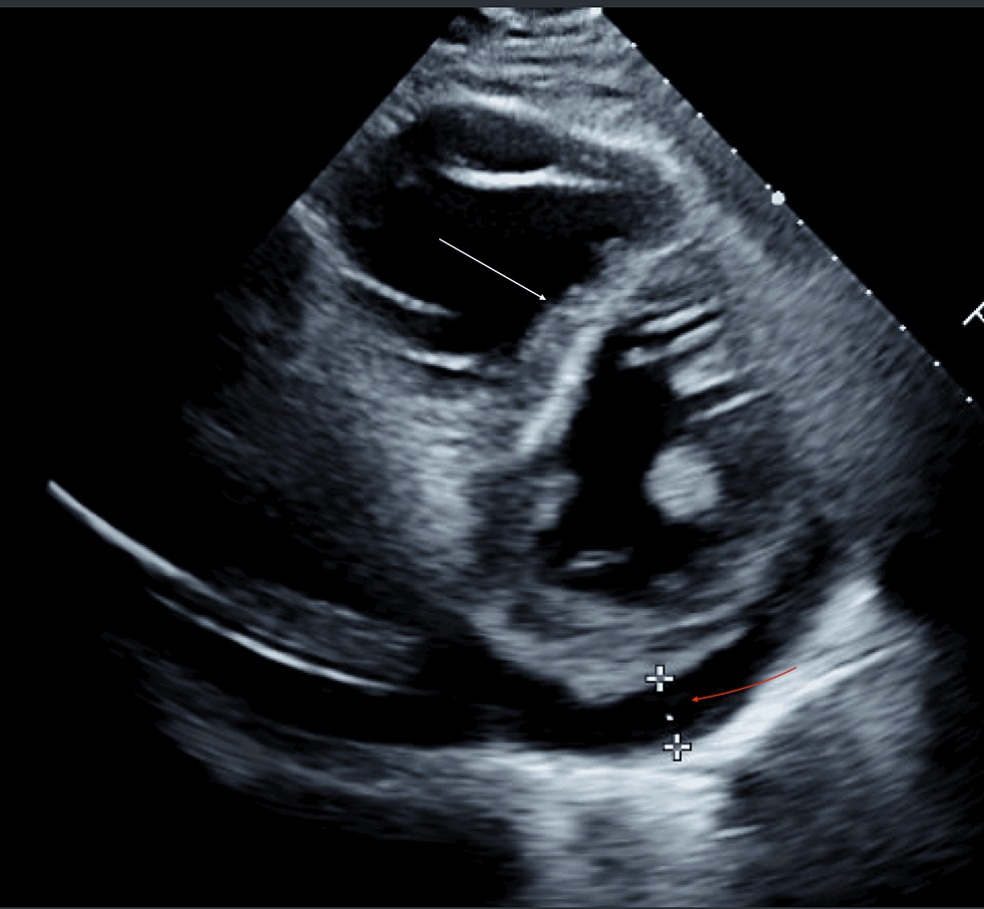
Short-axis transthoracic echocardiogram post-pericardiocentesis The image shows an enlarged RV with septal flattening (white arrow) consistent with RV strain not seen on the initial echocardiogram. There is also mild posterior pericardial effusion (red arrow). Interval resolution of the anterior pericardial effusion is seen RV: right ventricle

**Figure 4:**
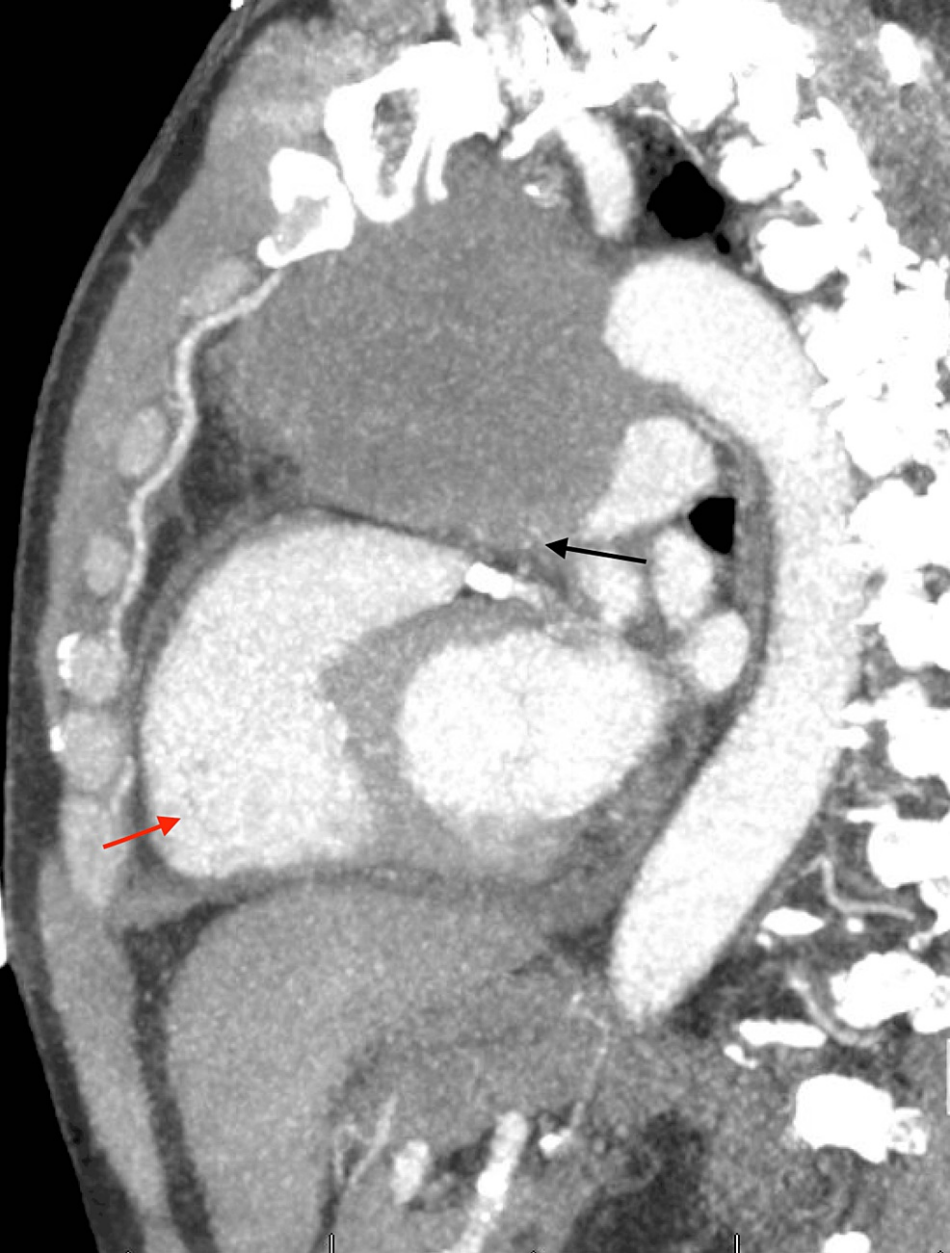
Chest CT post-pericardiocentesis CT with intravenous contrast mid-sagittal view showing large anterior mediastinal mass with significant main pulmonary artery compression (black arrow). There is evidence of right heart strain with an RV-to-LV ratio of approximately 1.5 (red arrow) CT: computed tomography; LV: left ventricle; RV: right ventricle

In light of the new malignancy diagnosis, the decision was made to proceed with pericardiocentesis instead of a pericardial window. A total of 600 cc of bloody fluid was removed. The pericardial drain was removed 24 hours later due to low output. After the pericardiocentesis, the patient developed recurrent episodes of atrial fibrillation with rapid ventricular response and hypotension that was managed with electrical cardioversion. He received volume resuscitation with 1 L lactated ringers with minimal improvement in his blood pressure. Due to the concern for an underlying cardiogenic shock, a repeat echocardiogram was performed. It revealed severe RV enlargement with an RV/LV ratio of 1.5 (Figure 3), suggesting RV strain. Pulmonary embolism was ruled out with a repeat CT chest with IV contrast, but it showed almost complete compression of the main pulmonary artery and signs of significant RV strain (Figure 4).

**Figure 5:**
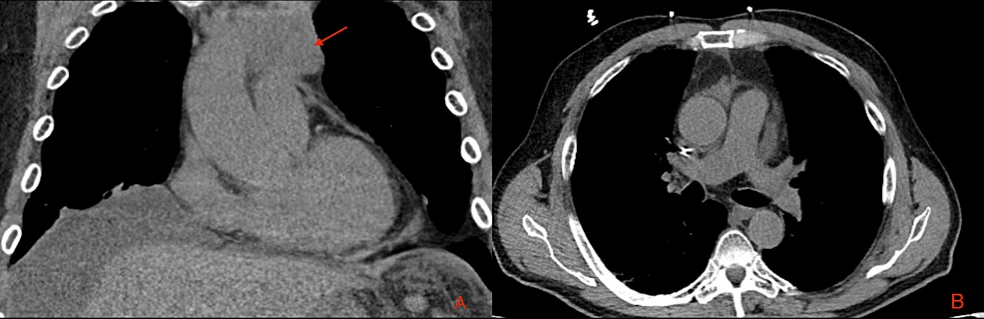
CT without contrast two weeks following chemoradiotherapy The images show an interval decrease in anterior mediastinal mass size (A) and resolution of main pulmonary artery compression (B) CT: computed tomography

The patient was not a candidate for surgical resection due to his hemodynamic instability as well as the direct tumor involvement of the great vessels making complete resection extremely challenging. He was emergently started on ionizing radiotherapy with 300 cGy spread over three fractions along with concurrent carboplatin/paclitaxel chemotherapy. It was followed by 30 radiation therapy fractions of 200 cGy each, along with the weekly carboplatin/paclitaxel regimen. The patient experienced an improvement in his hemodynamics, and the norepinephrine drip was stopped 48 hours after treatment initiation. Two weeks later, a follow-up chest CT scan showed the resolution of pulmonary artery compression and shrinkage in the anterior mediastinal mass (Figure 5). He was discharged home in stable condition on maintenance pembrolizumab immunotherapy. However, he was readmitted with ascites six months after his initial presentation and was diagnosed with peritoneal carcinomatosis. The patient elected to be discharged to home hospice care, and he expired nine months after the malignancy diagnosis.

## Discussion

The RV is embryologically derived from the second pharyngeal pouch. Compared to the LV, which has three distinct muscle fiber layers, the RV depends on the superficial (circumferential) fibers and deep longitudinal fibers [8]. It contracts in two planes: longitudinal from base to apex and horizontal from sidewall to septum. Due to its structure, the RV is extremely sensitive to pressure changes.

RV strain has been described in multiple cases of mediastinal tumors, either from direct compression [8] or secondary to increased afterload due to invasion of pulmonary vasculature as a part of the “mediastinal syndrome” [9]. A mediastinal syndrome refers to a group of disorders that causes complex hemodynamic imbalances from external compression of mediastinal structures, which require careful titration of cardiopulmonary support. Any hemodynamic imbalance in these patients can lead to what is known as the “pericardial decompression syndrome” or paradoxical hemodynamic instability [10,11]. Pericardiocentesis itself can exacerbate RV failure secondary to pericardial decompression syndrome (PDS). A study of 35 cases of PDS revealed that RV failure comprises around 9% of the cohort; the highest risk occurs in the first 48 hours after pericardiocentesis [12].

The pathophysiology of PDS occurs secondary to a rapid increase in preload and afterload. The preload increases due to rapid venous return. Moreover, increased pulmonary vascular flow can add to the afterload [13]. Also, V/Q mismatch occurs from airway compression in the setting of the mediastinal syndrome, and subsequent hypoxic vasoconstriction further adds to the former. Secondly, the sudden increase in end-diastolic volume leads to a mismatch in ventricular outputs due to the phenomenon of ventricular interdependence seen with increased RV/LV ratio [14,15,16]. This was particularly relevant in our case; the pericardial fluid compressing the RV may have maintained a narrower pressure gradient between the RV and the increased afterload from the tumor-acquired pulmonary artery compression. Pericardial drainage through pericardiocentesis abolished this gradient, creating a sudden increase in the RV end-diastolic volume and the pressure gradient across the pulmonic valve. The previously high afterload due to compressive pulmonary hypertension was likely worsened by the volume overload in the RV secondary to PDS. Our patients' hemodynamics stabilized after emergent chemoradiation and tumor shrinkage seen on imaging. The resolution of pulmonary artery compression reduced the afterload, restoring a physiologic pressure gradient across the pulmonic valve.

## Conclusions

The etiology of RV strain is broad. Mediastinal neoplasms with mass effect on the pulmonary artery and subsequent RV failure require rapid identification and prompt initiation of treatment. Clinicians should have a high degree of awareness and suspicion of PDS as a possible cause of RV failure. Careful monitoring and titration of hemodynamic cardiovascular parameters need to be implemented after pericardiocentesis if a mediastinal tumor is externally compressing the main pulmonary artery.
